# Repeatability and Predictability of Calf Feeding Behaviors—Quantifying Between- and Within-Individual Variation for Precision Livestock Farming

**DOI:** 10.3389/fvets.2022.827124

**Published:** 2022-03-31

**Authors:** Charles Carslake, Francesca Occhiuto, Jorge A. Vázquez-Diosdado, Jasmeet Kaler

**Affiliations:** School of Veterinary Medicine and Science, University of Nottingham, Sutton Bonington Campus, Leicestershire, United Kingdom

**Keywords:** feeding behavior, health and welfare, dairy calves, precision livestock farming, animal personality

## Abstract

Individual calves show substantial between- and within-individual variation in their feeding behavior, the existence and extent of which are not fully researched. In this study, 57,196 feeding records, collected by a computerized milk feeder from 48 pre-weaned calves over 5 weeks, were collated and analyzed for individual differences in three different feeding behaviors using a multi-level modeling approach. For each feeding behavior, we quantified behavioral variation by calculating repeatability and the coefficient of variation in predictability. Our results indicate that calves differed from each other in their average behavioral expression (behavioral type) and in their residual, within individual variation around their behavioral type (predictability). Feeding rate and total meals had the highest repeatability (>0.4) indicating that substantial, temporally stable between-individual differences exist for these behaviors. Additionally, for some behaviors (e.g., feeding rate) calves varied from more to less predictable whereas for other behaviors (e.g., meal size) calves were more homogenous in their within-individual variation around their behavioral type. Finally, we show that for individual calves, behavioral types for feeding rate and total meals were positively correlated which may suggest the existence of an underlying factor responsible for driving the (co)expression of these two behaviors. Our results highlight how the application of methods from the behavioral ecology literature can assist in improving our understanding of individual differences in calf feeding behavior. Furthermore, by uncovering consistencies between individual behavioral differences in calves, our results indicate that animal personality may play a role in driving variability in calf feeding behavior.

## Introduction

Repeated measures of livestock behavior are currently available to researchers and farmers thanks to the increasing sophistication and availability of sensor technologies (1–3). This has opened opportunities to continuously observe and analyze behavior at the level of individual farm animals. Such individualized monitoring can improve management, for example, by improving heat detection in dairy cattle (4). In addition, numerous technologies are being developed that may assist in detecting ill health in livestock by detecting changes in behavior that precede or occur alongside clinical disease [e.g., (5–7)]. Sensors could also be used to quantify potential indicators of positive welfare, such as play behavior in calves (8). However, different individuals tend to behave differently. Individuals may differ in their average behavioral expression (e.g., can be more or less active) or may differ in the degree to which their behavior varies around their respective means (9, 10). Where present, this variability in behavior may have important implications for behavioral monitoring since failure to account for normal, intrinsic variation (i.e., treating all animals the same) could result in false inferences and mislabeling, and hence impede successful algorithm development. In addition, the underlying behavioral tendencies that drive contextually and temporally consistent between-individual differences is termed animal personality (11). Differences in farm animal personality have implications for health, welfare, and productivity (12–14). Improving our understanding of the existence and extent of individual variation in behavior is an essential first step to enable researchers to exploit the potential of individualized behavioral monitoring.

The advancement of methods and measures in behavioral ecology offers a valuable opportunity to assess individual differences in behavioral expression [e.g., (15–18)]. These methods use repeated measures of animal behavior in a multilevel modeling framework allowing the calculation of behavioral measures that are statistically defined (19). For example, an individual's behavioral type corresponds to the value of its random intercept and, respectively, the individual's position upon a behavioral spectrum (16). The measure repeatability indicates the proportion of within- and between-individual variation in a behavior that can be explained by differences between individuals (20). Another measure is predictability, which quantifies the degree to which behavioral observations vary around an individual's average behavior or behavioral type (10). This approach is especially relevant for high-precision observations with low measurement error, where residual variation is assumed to be mainly systematic and biologically meaningful. Multivariate mixed modeling approaches build on this approach by allowing behavioral types and predictability, as well as correlations between these measures, to be estimated within a single statistical framework (18). Where correlations between these exist, these are termed behavioral syndromes the uncovering of which can improve our understanding of how different behaviors are related (21).

Monitoring feeding behavior in livestock is important for assessing productivity and evaluating health and welfare (22). Within dairy farms, the increasing use of computerized milk feeders for pre-weaned calves means that a wealth of data detailing calf feeding behavior is readily available. A large body of literature exists that harnesses these data to explore a variety of questions, such as the effect of different feeding regimes, changes in behavior that occur prior to ill health and differences in feeding behavior between personality types (23–25). While significant between-individual variation in feeding behavior has been reported in calves (26, 27), no studies have employed a quantitative approach to explore within and between-individual variability. Quantifying the repeatability of different feeding behaviors in calves could assist in characterizing consistent inter-individual differences in behavioral variation. Quantifying the coefficient of variation in predictability for each behavior could help identify behaviors for which calves differ in their residual intra-individual variation around their behavioral type. Correlating measures of behavioral variation among individuals gives us new insights into how these measures may be related. Such a quantitative analysis could form the basis of future work exploring the phenotypes of calves based on their feeding behavior.

In this study, we used repeated measures of feeding behavior obtained from a computer-controlled automatic milk feeder to quantify individual differences in feeding behavior in pre-weaned dairy calves using a multivariate multilevel modeling approach. First, we quantified individual variation in the average expression of behavioral traits by calculating the behavioral type for each calf and repeatability for different feeding behaviors. Second, we quantified differences in predictability by calculating residual intra-individual variation (rIIV) for each individual and the coefficient of variation in predictability for each behavior. Finally, we report the correlations between behavioral types and rIIV estimates.

## Materials and Methods

### Data Collection

#### Calf Recruitment

The study was conducted at the Centre for Dairy Science Innovation at the University of Nottingham, UK. All calves enrolled in the study were born at the farm between March 26, 2021 and August 29, 2021. There were 64 calves enrolled in the study; all calves were Holstein Friesian and female. This study used data collected by a computerized milk feeder (Forster-Technik Compact Smart) during the first 35 days of a period of group housing which took place as part of routine calf management. There were 16 calves per group and data were collected for four groups (cohorts) of calves.

#### Calf Management

##### Housing

Calf housing consisted of two stages: first, a period of pair housing (i.e., two calves per pen) followed by a period of group housing (16 calves per pen). At the first stage, calves were removed from the dam within 4 h of birth and housed in a straw-bedded pen (3 m × 2 m) in pairs (the two calves closest in age were paired together). Each pair had access to a feeding station which was equipped with a teat and operated by a computerized milk feeder. The feeding stations were ~1 m × 0.5 m and equipped with sides but there was no back gate to prevent displacements at the feeder. Each computerized milk feeder operated four feeding stations.

The second stage of housing commenced once 8 pairs of calves (i.e., 16 calves) reached a minimum of 21 days old. These calves were then grouped together in a large straw bedded pen (6 m × 12 m). Throughout this group housing period, each group of 16 calves had access to a single milk feeding station (i.e., 1 teat per 16 calves). The feeding station was not equipped with a back gate and there was one computerized feeder which operated two feeding stations (i.e., it fed 32 calves split between two pens). Data collection for this study took place during the first 35 days of this second stage (i.e., the period of group housing) only.

##### Feeding and Colostrum

Within 2 h of birth, calves were fed 4 l of pasteurized colostrum as per farm protocols. All colostrum is checked for quality using a colostrometer (a hydrometer that estimates IgG density by measuring colostrum density). The colostrum protocol is routinely evaluated by screening subsets of calves for failure of passive transfer by checking total serum protein levels using a refractometer (failure of passive transfer is defined as serum total protein levels < 5.0 g/dL). Post-colostrum, calves are fed pasteurized transition milk (4 l twice daily) from a bucket equipped with a teat until 2 days of age. At 2 days of age, farm staff gently guided calves toward the feeding station present in the pen where the calves were shown the teat and fed a milk allowance by the computerized feeder. From this point, the calves learned, with occasional assistance by farm staff, to feed from the teat present in the feeding station.

Each calf was equipped with an RFID ear tag and each feeding station was equipped with an RFID reader. This allowed the computerized milk feeder to recognize the identity of each calf when present at the feeder, mix a portion of milk replacer from milk powder and warm water (130 g/l), and dispense the calf a milk allowance. All calves were fed the same milk replacer (Milkivit Energizer ECM, Trouw Nutrition GB) for the entire milk-feeding period. Upon recognition of the calf's RFID ear tag at the station, if the calf is due a milk feed it is dispensed a maximum of 2 l per feed. Once the calf has drank the allowance (called entitlement), the next entitlement will not be dispensed another for another 2 h. If the calf does not drink the whole 2-l entitlement, the remaining is kept available for the next 2 h after which time the entitlement restarts at 2-l.

The computerized feeder was preprogrammed to allocate each calf a total daily milk allowance which renewed from 00:00:00 every morning. The daily allowance fed by the computerized feeder started at 6 l at 2 days old and increased daily in line with age, reaching 8 l at 5 days old. From 8 days old, the daily allowance increased daily reaching a plateau of 10 l from 40 days old. During the second stage (i.e., group housing), all calves, regardless of age, were fed 10 l daily for the 35 days following the move to the group pen. After this, the allowance was reduced by 400 ml/day. This meant that 25 days later (i.e., after 60 days in the group pen) the milk allowance was reduced to 0. Calves had *ad libitum* access to concentrates (FiMLAC Sweet Start Pellets), chopped straw, and water throughout.

##### Health Monitoring and Vaccines

A veterinary surgeon manually inspected all calves twice weekly for any signs of ill health using the Wisconsin calf health scoring system (28). This system combines rectal temperature and weighted scores of clinical signs (i.e., nasal discharge) to detect ill health in calves. In addition, farm staff visually inspected calves twice daily. Any calves with signs of ill health were treated according to the farm protocols and advice from the farm's veterinary surgeon (e.g., antibiotic and anti-inflammatory for respiratory disease, oral rehydration therapy for mild diarrheal disease). Calves were vaccinated with a vaccine against respiratory disease (Rispoval RS+Pi3 IntraNasal; Zoetis) at 9 days of age.

### Data Acquisition and Selection

The computerized milk feeder used in this study logs each visit a calf makes to the feeder on a software program. Data recorded by the feeder include calf identity, date, time the calf entered the feeder, time the calf left the feeder, if the calf was entitled to a milk feed, feed consumption, and feeding rate for each visit. A new visit (row) was created whenever the RFID reader loses and then regains contact with an RFID tag.

Data from the computerized milk feeder for the group-housed calves were downloaded and combined. The first 2 days of group housing were excluded to allow a period of acclimatization to the new environment and feeder. The subsequent 33 days of group housing were included in this analysis to ensure that all calves were on a level feeding plane prior to any reduction in milk allowance. We excluded all calves that were categorized as sick by our health scoring from our analysis (Wisconsin score > = 5; *n* = 16) to ensure no clinically diseased calves were included in our study (Table 1) (28). Due to technical problems (failure to save data onto an SD card) and management procedures (e.g., cleaning of pens) a maximum of 10 days and a minimum of 3 days were excluded for each group. The remaining data corresponded to 57,196 rows.

**Table 1:** Number of calves included in analysis per cohort as per the inclusion criteria detailed in the methods and age of calves at start of data collection.

	**Cohort 1**	**Cohort 2**	**Cohort 3**	**Cohort 4**	**Overall**
**Calves**					
Calves excluded	4	6	3	3	16
Calves included	12	10	13	13	48
**Age at trial start (days)—included calves only**					
Mean	34.4	31.8	41.0	39.9	40.0
Min	21	23	26	26	21
Max	48	39	54	50	54

### Data Processing

#### Meal-Based Criterion

All preprocessing and analysis were undertaken in R software (version 4.1.0) (29). We grouped visits to the feeder by the same calf that were closely clustered in time (<100 s) into a single meal. Meal based estimates better characterizes calf feeding behavior compared to studying visits alone, since they allow the calculation of interesting characteristics such as whether each meal was associated with a milk feed, whether the calf was entitled to milk feed, and total feed consumption during each meal. Clustering visits into meals requires the usage of a meal criterion. A meal criterion corresponds to a maximum time interval between the end of the same calf's visit to the feeder and the start of its next visit, to consider these visits as part of the same meal (30, 31). We used a simple method previously described in adult cattle where, for each calf, the interval between consecutive visits to the feeder is calculated, and its log-transformed distribution plotted (32). Visual inspection revealed three distributions with intersections of ~100 s and 1,600 s. Since we are interested in the quantifying returns to the feeder that could occur within the longer interval period of 1,600 s (~26 min), we chose the shorter period of 100 s as our meal criterion (see Supplementary Material for further detail).

#### Feeding Behaviors

For each calf and for each day of the group-housing period, we calculated variables to describe the calves' feeding behavior. These are detailed in Table 2. During this stage of the preprocessing a small number (*n* = 4) of non-sensical recordings (assumed measurement errors) were excluded.

**Table 2:** Definition of feeding behaviors used.

**Feeding behavior**	**Definition**
Total meals	Daily sum of all meals. This variable includes meals where the calf is entitled to a milk feed and meals where the calf is not entitled to a milk feed.
Meal size	Mean daily meal size calculated from meals where the calf is entitled to milk and consumes a milk feed within the same meal. It corresponds to the quantity of milk consumed divided by the number of these meals.
Feeding rate	Mean daily feeding rate calculated by the feeder and reported for each visit. We only included visits where the calf is entitled to a milk feed and consumes milk.

### Analysis

All statistical analyses were carried out using R software (version 4.1.0) (29). Code for the figures was adapted from Hertel et al. (15, 16).

#### Multivariate Double Hierarchical Generalized Linear Model

We ran a single multivariate double-hierarchical generalized linear model (DHGLM) with the three feeding behaviors (feeding rate, total meals, and meal size) as response variables using the “brms” package in R (33). A DHGLM was chosen as it includes two parts; a “mean” and a “dispersion” part. The mean part of the model is focused on the estimation of individuals' means while the dispersion part is concerned with modeling the residual variance (i.e., the variation around the mean). The model can be written as Equations 1, 2 for mean and Equations 3–6 for the dispersion part of the models (17).


(1) $$\begin{array}{l} Y_{i}=X\beta +Z\alpha +\varepsilon \end{array}$$

(2) $$\begin{array}{l} \alpha \sim N\left(0,\;{I_{m}\sigma }_{\alpha }^{2}\right), \end{array}$$

(3) $$\begin{array}{l} \varepsilon \sim N(0,\;Diag\{\sigma_{\varepsilon }^{2}\}), \end{array}$$

(4) $$\begin{array}{l} log\left(\sigma_{\varepsilon }\right)=\eta_{\text{d}}, \end{array}$$

(5) $$\begin{array}{l} \eta_{d}=\;X_{d}\beta_{d}+Z_{d}\alpha_{d}, \end{array}$$

(6) $$\begin{array}{l} \alpha_{d}\sim N\left(0,\;{I_{m}\omega }_{\sigma_{d}}^{2}\right) \end{array}$$

In the model α represents individual-specific random effect variation, *Y*_*i*_ represents the response variables (feeding rate, total meals, and meal size), *X* represents the fixed effects, *Z* the random effects, residual deviations from the prediction are represented by ε. Terms *X*_*d*_ represent the fixed effects for the dispersion part of the model, *Z*_*d*_α_*d*_ the random effects component of the dispersion, and $\omega_{\sigma }^{2}$ represents the dispersion model hyperparameter. Between-individual random effects of variance (α) are assumed normally distributed as well as α_*d*_and individual-specific residual standard deviations ($\sigma_{\varepsilon }^{2}$) are assumed to follow a log-normal distribution. For both the mean and dispersion parts of the model, we included age at grouping, day number, and their interaction term as fixed effects. We included individual Calf ID and cohort as random effects. All distributions were specified as Gaussian. To capture a Gaussian posterior distribution, we log transformed the variable total meals. Meal size was transformed using an ordered quantile normalization which was selected using the “bestNormalize” package in R (34). Feeding rate was normally distributed. All variables were scaled after transformation (mean = 0; SD = 1).

We used uninformative priors for both fixed and random effects. We ran four chains for 12,000 iterations, a warmup of 4,000 iterations, and a thinning interval of 4. Model diagnostics indicated satisfactory convergence with R < 1.01 and effective sample sizes > 400. Posterior predictive checks indicated that the underlying Gaussian distribution was satisfactorily captured.

#### Repeatability Estimates

Repeatability ***Rpt*** was defined as the variance among group means (i.e., the variance explained by differences between individual calves) ***VCalfID*** over the sum of the variance explained by differences between individual calves and the residual variance ***Vresidual*** that reflects the variance within individuals. In our model ***Vresidual*** corresponds to the population intercept of the residual model and was converted to a variance by taking its exponent and squaring the resulting value (15).


$$\begin{array}{l} \text{Rpt}=\text{VCalfID}/(\text{VCalfID}+\text{Vresidual}) \end{array}$$

***Rpt*** values are between 0 and 1. Higher values of ***Rpt*** for a behavior indicate the population is composed of individuals that behave consistently differently from each other whereas low values indicate that individuals are more similar. We describe our results for ***Rpt*** as higher or lower with reference to a meta-analysis which summarized 759 estimates of repeatability from 114 studies and indicated that the mean level of repeatability was 0.37 [0.36–0.38] (20).

#### Coefficient of Variation in Predictability Estimates

For each behavior, we used the dispersion part to estimate the residual intra-individual variation (rIIV), after controlling for fixed effects, for each individual calf (17). Calves with higher rIIV are less predictable (i.e., have greater variation around their means) than calves with lower rIIV. For each behavior, between-calf variation in rIIV was quantified by calculating the coefficient of variation in predictability ***CV***_***P***_. This measure quantifies the population-level variation in predictability (17). In the equation below, the term ω***2*** represents the dispersion model hyperparameter (the estimate for individual differences in residual variance) which can be extracted from the DHGLM.


$$\begin{array}{l} CV_{P}=\surd (\text{exp}(\omega^{2}-1) \end{array}$$

***CV***_***P***_ values are between 0 and 1. Higher values of ***CV***_***P***_ for a behavior indicate the population is composed of individuals that vary in their rIIV (i.e., a mixture of predictable and unpredictable individuals), whereas a lower value for ***CV***_***P***_ indicates that individuals express similar levels of behavioral variation around their respective behavioral types. We describe our results for ***CV***_***P***_ as higher or lower with reference to a meta-analysis which summarized 64 estimates of ***CV***_***P***_ from 39 studies indicated that behavioral traits had mean ***CV***_***P***_ of 0.27 [0.22, 0.33] (35).

#### Correlations Between Variance Components

In addition to calculating behavioral types (the mean behavior after controlling for fixed effects) and the rIIV for each behavior the multivariate DHGLM computes the correlations between these estimates. Since these behavioral types and rIIVs are estimated in the same framework, any uncertainty around estimates of the mean is carried forward into the correlations between these. This approach allows us to test for correlations between behavioral types and predictability estimates (i.e., feeding rate behavioral type and total meals behavioral type) while avoiding the potential pitfall of inflated *p*-values that can occur when uncertainty around model estimates is ignored (18).

## Results

### Calf Feeding Behaviors Have Different Repeatability

The degree to which individuals differ from each other, as a proportion of within and between individual variation, varied by behavior and is reported in Table 3. Table 3 reports repeatability after controlling for the effect of age, day number, and cohort (adjusted repeatability) of the feeding behaviors monitored in this study. Repeatability was highest for feeding rate followed by total meals. Repeatability was considerably lower for meal size.

**Table 3:** Mean, median, inter-quartile range, repeatability, and coefficient of variation in predictability for total meals, feeding rate, and meal size.

	**Feeding rate (ml/min)**	**Meal size (ml)**	**Total meals (*n*)**
Mean	831	1,989	10.1
Median	835	2,045	9
IQR	770–901	1995–2054	7–12
**Repeatability**			
Estimates	0.50	0.03	0.42
CrI	0.32–0.68	0.00–0.06	0.30–0.55
**Coefficient of variation in predictability**			
Estimates	0.27	0.07	0.13
CrI	0.21–0.37	0.00–0.13	0.06–0.21

*IQR and CrI correspond to interquartile range and credibility interval, respectively*.

**Figure 1:**
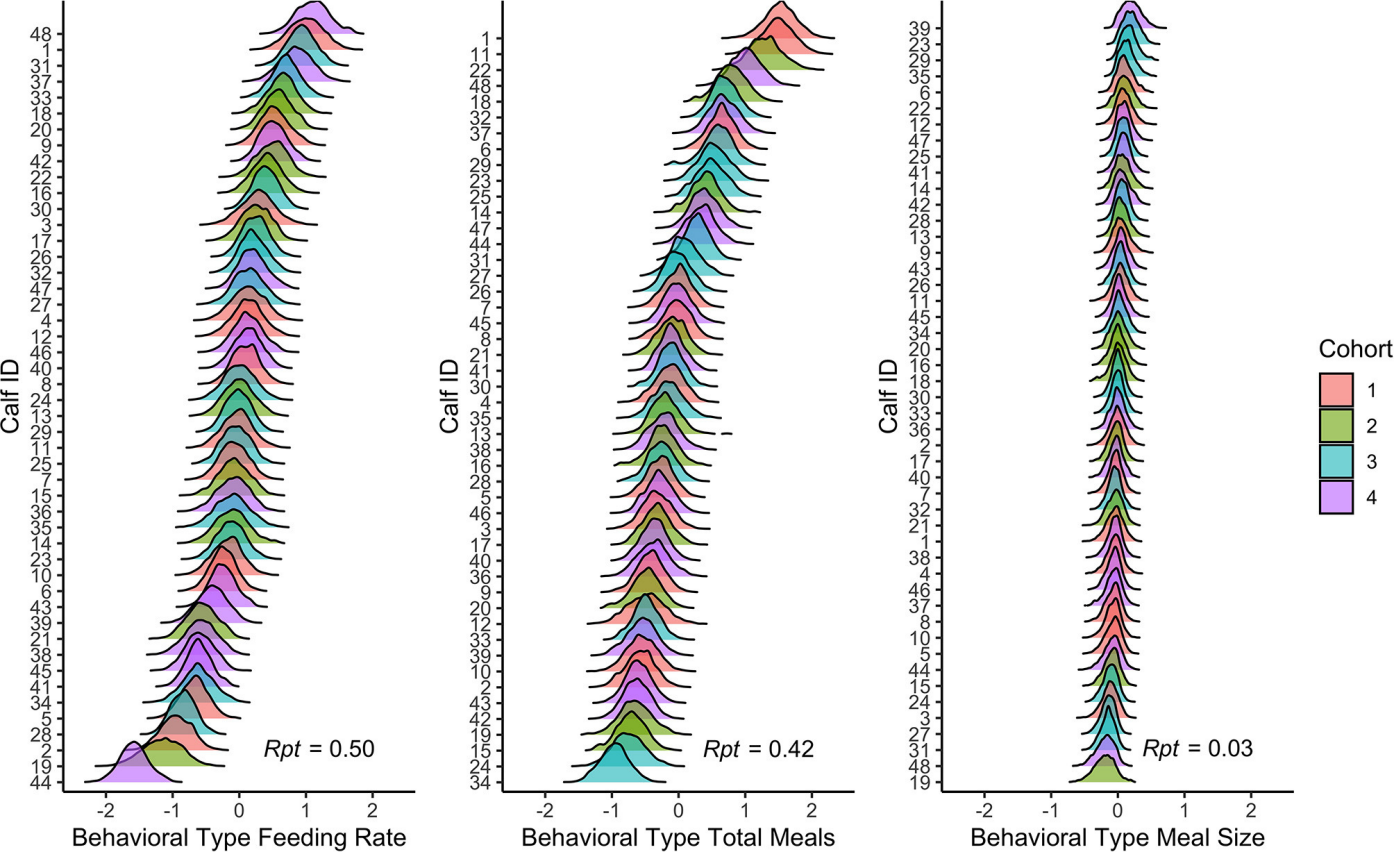
Posterior distributions of behavioral types from the double hierarchical mixed models for feeding rate, total meals, and meal size for each calf. The models controlled for between-individual differences in age, day number, and cohort. Variables are Z-transformed (mean = 0, SD = 1). The ridges indicate the posterior 95% credible interval, and the different colors correspond to the different cohorts. Repeatability (Rpt) is reported in the bottom right corner of each panel.

Figure 1 illustrates the concept that different behaviors have different repeatability by plotting behavioral type estimates on a spectrum from low to high. Behaviors are Z-transformed (mean = 0, SD = 1) to facilitate comparison. As a proportion of total behavioral variation, behaviors with high repeatability (e.g., feeding rate) had greater between-individual differences in behavioral type than behaviors with low repeatability (e.g., meal size).

### Calf Feeding Behaviors Have Different Coefficients of Variation in Predictability

For each behavior, the degree to which calves differ in terms of their predictability, i.e., the degree to which individual calves differ in their residual intra-individual variation around their respective means, is quantified by the coefficient of variation in predictability in Table 3. The coefficient of variation in predictability was highest for feeding rate.

**Figure 2:**
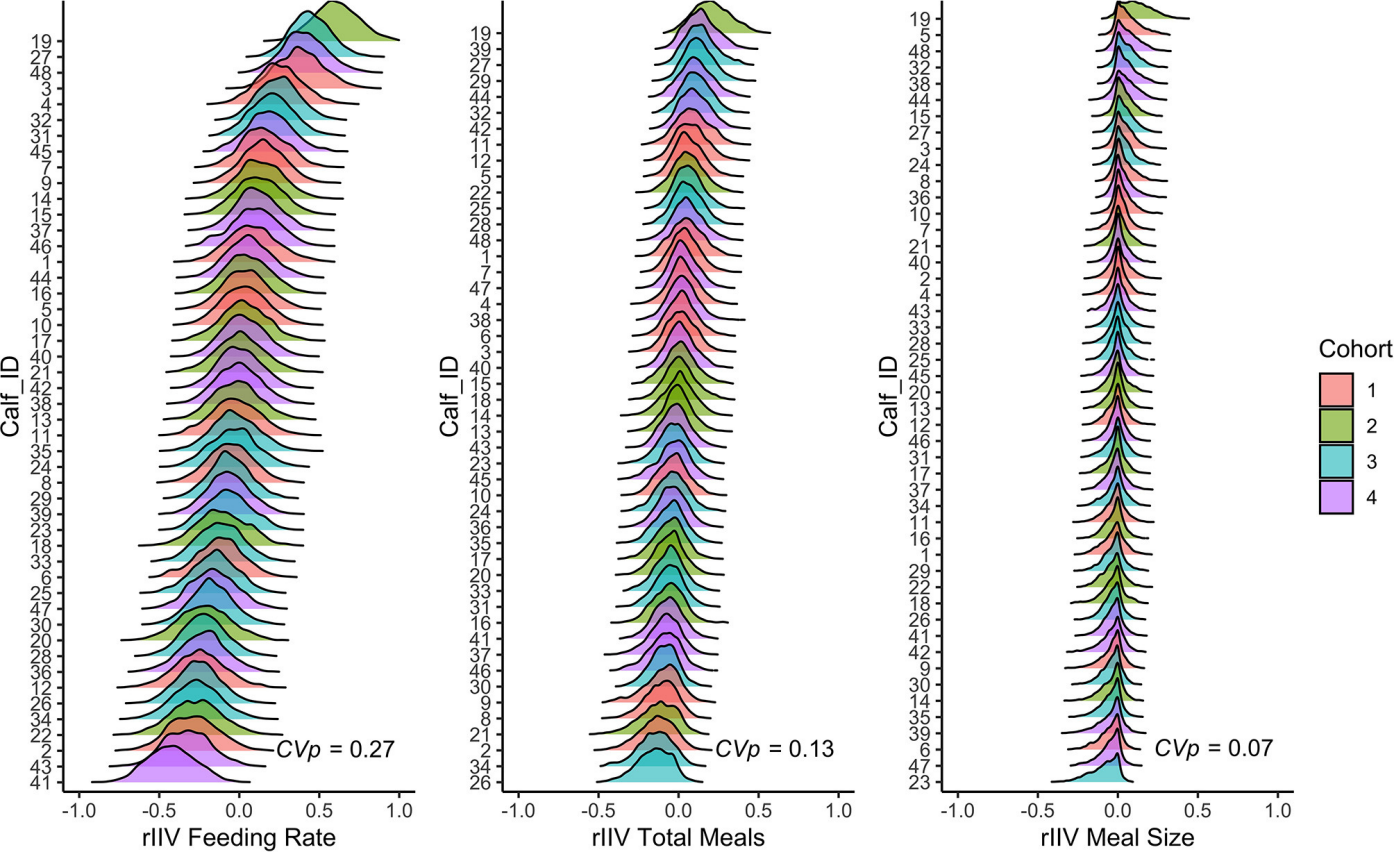
Posterior distributions of residual intraindividual variation (rIIV) from the double hierarchical mixed models for feeding rate, total meals, and meal size for each calf. The models controlled for between individual differences in age, day number, and cohort. Variables were Z-transformed (mean = 0, SD = 1). The ridges indicate the posterior 95% credible interval, and the different colors correspond to the different cohorts. The coefficient of predictability (CVp) is reported in the bottom right corner of each panel.

Less predictable individuals have high variance around their respective behavioral types (high residual intra-individual variation), while more predictable individuals have low residual intra-individual variation (rIIV). Figure 2 illustrates the concept that different behaviors have different coefficients of predictability by plotting rIIV estimates on a spectrum from low to high. Behaviors are Z-transformed (mean = 0, SD = 1) to facilitate comparison. Behaviors with higher coefficients of predictability (e.g., feeding rate) have greater between individual differences in rIIV than behaviors with lower coefficients of predictability (e.g., total meals and meal size).

### Calves' Feeding Rate Behavioral Type Was Correlated With Their Total Meals Behavioral Type

**Figure 3:**
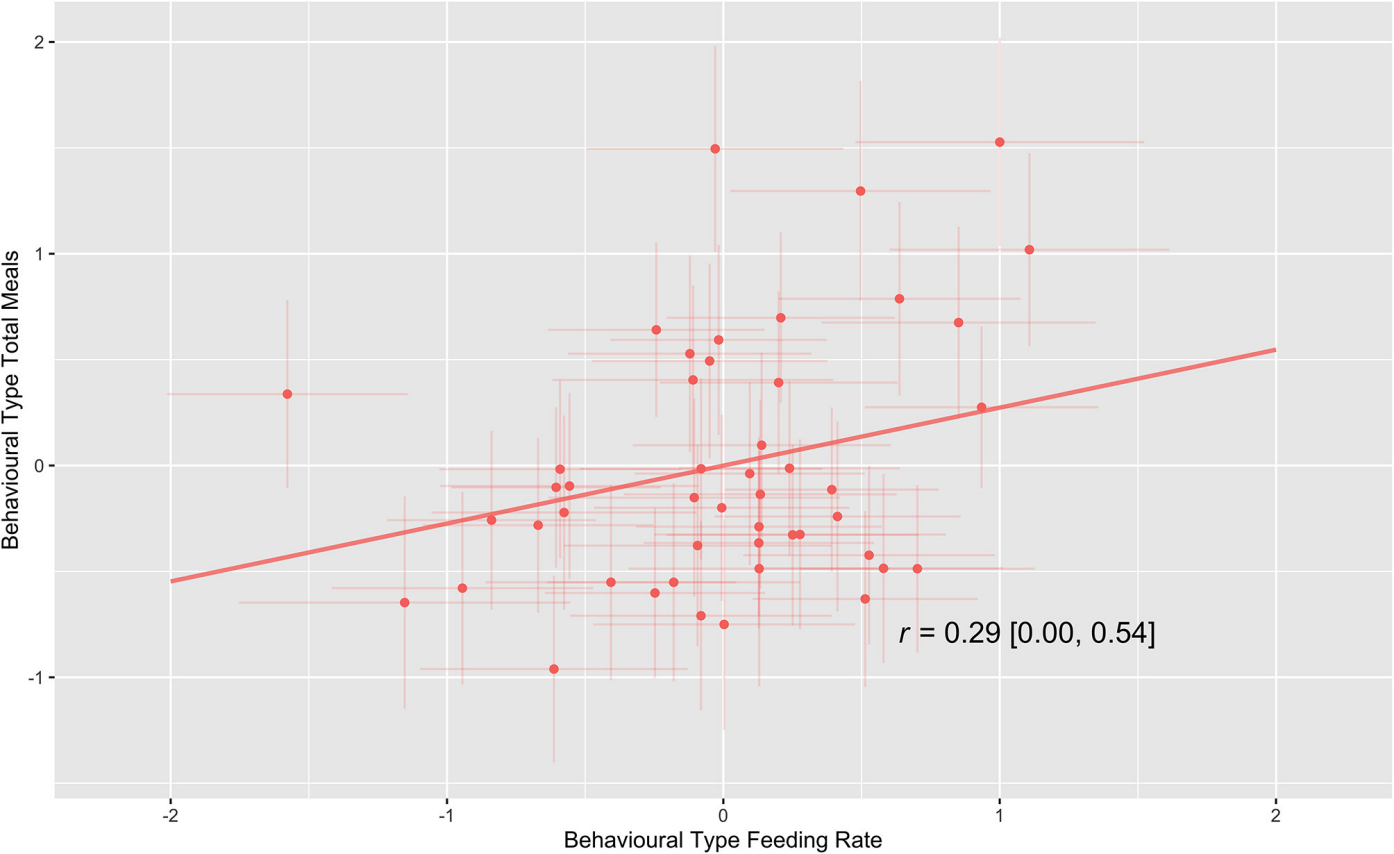
Visual representation of the line of best fit of the among-individual correlation (r) between feeding rate behavioral type and total meals behavioral type. Posterior means and 95% credible intervals of point estimates for each calf are shown.

Estimates of our multivariable double hierarchical mixed model indicate that there was a significant positive linear correlation (r = 0.29 [0.00–0.54]) between individual calves' behavioral types for feeding rate and total meals as is shown in Figure 3. This result shows that calves that drank faster had more meals, and calves that drank slower had fewer meals. No correlations were present between individual calves' predictability (rIIV) and behavioral types for the other behaviors, these are detailed in the Supplementary Materials.

## Discussion

Our study is the first to quantify individual differences in calf feeding behavior at the between- and within-individual levels. Behavioral type refers to individual's average behavioral expression and predictability refers to its within-individual variation around its behavioral type. For each behavior, individual calves can be situated both on a spectrum of behavioral types (low to high) and a spectrum of predictability (low to high). The repeatability (the degree to which these individual differences in behavioral type explained the total variation) varied by behavior and was greatest for feeding rate and lowest for meal size. The coefficient of variation in predictability (the degree to which individuals differed from each other in their predictability) was greatest for feeding rate. Interestingly, for feeding rate and total meals, our results revealed a within-individual correlation between behavioral types, suggesting a behavioral syndrome for this behavior. This result indicates that calves may be placed on an underlying axis with calves that drink more quickly and visit the milk feeder more frequently at one end to calves that drink more slowly and visit the feeder less frequently at the other.

Repeatability was highest for feeding rate indicating that calves had consistently different feeding rates. High levels of between-individual variation in feeding rate have been noted in calves (26), and between-individual variation in feeding rate has been examined in rats (36), pigs (37), and goats (38, 39). Our results support the idea that feeding rate is a robust feature of the individual in a variety of species (40). The degree to which calves differed from each other in their predictability, quantified by the coefficient of variation in predictability, was also greatest for feeding rate. Our results suggest that calves with predictable and calves with unpredictable feeding rates coexist.

The existence of between-individual variation in behavioral types and in predictability has implications for studies that aim to detect ill calves by use of their feeding behavior. The fact that calves differ in their behavioral type for some feeding behaviors (i.e., feeding rate, total meals) means that algorithms aiming to detect abnormalities using this behavior will need to account for different behavioral types for each calf. This can be achieved with approaches such as cumulative sum, which aims to detect abrupt change from an individualized baseline (41, 42). However, our results also suggest that for behaviors with relatively higher coefficients of variation in predictability, such as feeding rate in our study, it may also be necessary to allow different individuals differing levels of variation around the mean. This may be necessary to avoid flagging unpredictable but otherwise healthy individuals as abnormal.

We can also consider how these results may relate to the study of animal personality. Animal personality is defined as underlying behavioral tendencies that drive contextually and temporally consistent differences between individuals' behavioral expression (11, 43). The study of animal personality is based on the characterization of between individual behavioral variation and behaviors with high repeatability, such as feeding rate in our study, are particularly useful to the study of animal personality (20). Indeed, one study in calves found that individuals that had lower feeding rates were slower to interact with a novel object (more fearful) (44). Furthermore, in other species, an individual's boldness has been linked with its predictability (10). Bolder and more risk-taking individuals are more predictable, possibly, because they are less likely to change their behavior in response to micro-environmental perturbations (45–47). Between-individual variation in predictability is an axis of behavioral variation that has not previously been explored in calves. Recent work in adult cattle indicates that those individuals with lower within-individual variation (i.e., more predictable) may be better able to cope with their environment (48, 49). Future research could explore how feeding behavior behavioral types and predictability estimates in calves are related to personality traits as well as medium- and long-term health and production outcomes.

Repeatability was lowest for meal size. This result contrasts with one study in goats where meal size had high repeatability (38). Calves in our study were fed a restricted feeding plan using the automatic feeder, which allocated a maximum meal size of 2 l and restricted total daily intake to 10 l. In addition, there were 16 calves present for a single feeder which is likely to have led to some competition between calves for access to the feeder (50). These constraints may have prevented calves displaying their preferred meal size and number of meals, especially if their preference exceeded the limits imposed or if they were frequently displaced. Nonetheless, there was evidence for moderate levels of repeatability for total meals. The variable total meals is the sum of meals with entitlement and meals without entitlement. Between-individual variability in the number of meals with entitlement is likely to be limited by the feeder due to the restrictions on total meals size and total daily intake. No such limits are present for meals without entitlement indicating that the between individual variation in total meals could be largely driven by meals without entitlement. Fewer visits to the feeder without entitlement are associated with ill health in dairy calves (24). Our results suggest that to use this behavior as a feature to predict ill health it may be necessary to first account for substantial between-individual variation in the number of visits to the feeder.

Results from our model show a positive and significant correlation between total meals and feeding rate behavioral types. This result indicates that calves that had higher behavioral types for feeding rate also had higher behavioral types for total meals. Among individual correlations between behavioral types of distinct behaviors have been termed behavioral syndromes, a concept that is used to support the idea that there are underlying traits that drive the expression of more than one behavior. For example, in animal personality research, behavioral syndromes are of interest as they may relate to an underlying personality trait that is responsible for the co-expression of suites of correlated behaviors (21). More broadly, the identification of behavioral syndromes is an important area of research that is helpful for understanding the (co)evolution of behaviors or behavioral specialization within a group (51). Indeed, it is possible that a behavioral syndrome exists between feeding rate and total meals because of an underlying factor that drives them both. For example, the paradigm of pace of life hypothesis developed in behavioral ecology situates individuals on an axis between fast and slow pace of life (52). Benefits of a fast pace of life include higher metabolism, growth, and earlier reproduction. However, there are associated costs, such as a shorter lifespan and reduced investment in immune function (53). Calves that have consistently higher feeding rates and higher meal frequencies could be those situated at the “fast” end of the continuum, while those that have lower feeding rates and meal frequencies could be at the “slow” end. Accordingly, using such data to phenotype individuals at a young age could enable management strategies such as individualized feeding plans or identify individuals that may benefit from smaller group sizes where there is less competition around the feeder.

A variance partitioning approach such as we present here provides a relatively simple and scalable way to explore individual variation in livestock behavior. It is important to note that our measures of repeatability and predictability were calculated over a relatively short period (33 days) and therefore represent estimates of short-term repeatability and predictability (54). Both short- and long-term intervals are required to assess the temporal stability of these behavioral traits and the correlations between them. In addition, while we included four different cohorts in our study over different times of year, all calves were housed in the same environment, were fed the same milk allowance, and each group had the same number of calves per feeding station. Future studies could vary these constraints to investigate the contextual consistency of the between-individual differences and correlations reported here, evidence of which would further support the hypothesis that the observed differences in feeding behaviors could be driven by differences in personality (55). Differences in personality have been correlated with health and production outcomes (12, 25). Future studies could also explore the relationship between the measures reported here and outcomes such as daily live weight gain or immune function. In this study we did not include birth weight; its impact could be explored in future studies. During our study, we only included clinically healthy calves, and we manually inspected the calves twice weekly using an industry standard scoring system in addition to twice-daily visual inspections (28). However, no gold standard observational test exists for the diagnosis of ill health in calves (56, 57). It is possible that calves with subclinical disease could have been included in our analysis. New technologies may improve the sensitivity of disease detection by continuously monitoring physiological parameters such as core body temperature, allowing studies to better control for the potential effect of subclinical disease (58).

The approach we present here could be expanded further by incorporating measures from sensors that can monitor other behaviors such as general activity and/or social interactions between calves. Such research could assist in improving our understanding of between individual variation in these behaviors and how different behavioral types and predictability estimates are related to each other. Finally, while quantifying between individual differences and exploring the relationships between measures of behavior allows us to hypothesize that personality traits could be driving among-individual differences, it may be necessary to employ different statistical approaches to test if these traits can be measured directly using farm technologies. One such approach could be structural equation models (59, 60), which could be used to estimate relationships between such hypothesized latent traits and observed variables such as feeding behaviors.

## Data Availability Statement

The raw data supporting the conclusions of this article will be made available by the authors, without undue reservation.

## Ethics Statement

The animal study was reviewed and approved by Ethics Committee of the School of Veterinary Medicine and Science, University of Nottingham (unique reference number 1481 150603).

## Author Contributions

JK, JV-D, FO, and CC: conceptualization and writing—review and editing. CC: formal analysis, data curation, and writing—original draft preparation. JK: resources, funding acquisition, and supervision. CC and FO: data collection. FO, CC, and JK: project administration. All authors contributed to the article and approved the submitted version.

## Funding

This research was funded by the Biotechnology and Biological Sciences Research Council, United Kingdom, project reference: BB/M008770/1.

## Conflict of Interest

The authors declare that the research was conducted in the absence of any commercial or financial relationships that could be construed as a potential conflict of interest.

## Publisher's Note

All claims expressed in this article are solely those of the authors and do not necessarily represent those of their affiliated organizations, or those of the publisher, the editors and the reviewers. Any product that may be evaluated in this article, or claim that may be made by its manufacturer, is not guaranteed or endorsed by the publisher.

## References

[1] Berckmans D. Precision livestock farming technologies for welfare management in intensive livestock systems. OIE Rev Sci Tech. (2014) 33:189–96. 10.20506/rst.33.1.2273 25000791

[2] Brown DD Kays R Wikelski M Wilson R Klimley AP. Observing the unwatchable through acceleration logging of animal behavior. Anim Biotelemetry. (2013) 1:20. 10.1186/2050-3385-1-20

[3] Cooke SJ Hinch SG Wikelski M Andrews RD Kuchel LJ Wolcott TG. Biotelemetry: a mechanistic approach to ecology. Trends Ecol Evol. (2004) 19:334–43. 10.1016/j.tree.2004.04.003 16701280

[4] Mottram T. Animal board invited review: precision livestock farming for dairy cows with a focus on oestrus detection. Animal. (2016) 10:1575–84. 10.1017/S1751731115002517 26608699

[5] Schillings J Bennett R Rose DC. Exploring the potential of precision livestock farming technologies to help address farm animal welfare. Front Anim Sci. (2021) 2:13. 10.3389/fanim.2021.639678

[6] Barker ZE Vázquez Diosdado JA Codling EA Bell NJ Hodges HR Croft DP. Use of novel sensors combining local positioning and acceleration to measure feeding behavior differences associated with lameness in dairy cattle. J Dairy Sci. (2018) 101:6310–21. 10.3168/jds.2016-12172 29705427

[7] Matthews SG Miller AL Clapp J Plötz T Kyriazakis I. Early detection of health and welfare compromises through automated detection of behavioural changes in pigs. Vet J. (2016) 217:43–51. 10.1016/j.tvjl.2016.09.005 27810210 PMC5110645

[8] Carslake C Vázquez-Diosdado JA Kaler J. Machine learning algorithms to classify and quantify multiple behaviours in dairy calves using a sensor: moving beyond classification in precision livestock. Sensors. (2020) 21:88. 10.3390/s21010088 33375636 PMC7795166

[9] Dingemanse NJ Kazem AJN Réale D Wright J. Behavioural reaction norms: animal personality meets individual plasticity. Trends Ecol Evol. (2010) 25:81–9. 10.1016/j.tree.2009.07.013 19748700

[10] Stamps JA Briffa M Biro PA. Unpredictable animals: individual differences in intraindividual variability (IIV). Anim Behav. (2012) 83:1325–34. 10.1016/j.anbehav.2012.02.017

[11] Réale D Reader SM Sol D McDougall PT Dingemanse NJ. Integrating animal temperament within ecology and evolution. Biol Rev. (2007) 82:291–318. 10.1111/j.1469-185X.2007.00010.x 17437562

[12] Koolhaas JM van Reenen CG Animal Behavior and Well-Being Symposium. Interaction between coping style/personality, stress, and welfare: relevance for domestic farm animals1. J Anim Sci. (2016) 94:2284–96. 10.2527/jas.2015-0125 27285906

[13] Proudfoot KL Weary DM von Keyserlingk MAG. Linking the social environment to illness in farm animals. Appl Anim Behav Sci. (2012) 138:203–15. 10.1016/j.applanim.2012.02.008

[14] Finkemeier MA Langbein J Puppe B. Personality research in mammalian farm animals: concepts, measures, and relationship to welfare. Front Vet Sci. (2018) 5:131. 10.3389/fvets.2018.00131 30003083 PMC6031753

[15] Hertel AG Royauté R Zedrosser A Mueller T. Biologging reveals individual variation in behavioural predictability in the wild. J Anim Ecol. (2021) 90:723–37. 10.1111/1365-2656.13406 33301175

[16] Hertel AG Niemelä PT Dingemanse NJ Mueller T. A guide for studying among-individual behavioral variation from movement data in the wild. Mov Ecol. (2020) 8:1–18. 10.1186/s40462-020-00216-8 32612837 PMC7325061

[17] Cleasby IR Nakagawa S Schielzeth H. Quantifying the predictability of behaviour: statistical approaches for the study of between-individual variation in the within-individual variance. Methods Ecol Evol. (2015) 6:27–37. 10.1111/2041-210X.12281

[18] Houslay TM Wilson AJ. Avoiding the misuse of BLUP in behavioural ecology. Behav Ecol. (2017) 28:948. 10.1093/beheco/arx023 29622923 PMC5873244

[19] Dingemanse NJ Dochtermann NA. Quantifying individual variation in behaviour: mixed-effect modelling approaches. J Anim Ecol. (2013) 82:39–54. 10.1111/1365-2656.12013 23171297

[20] Bell AM Hankison SJ Laskowski KL. The repeatability of behaviour: a meta-analysis. Anim Behav. (2009) 77:771–83. 10.1016/j.anbehav.2008.12.022 24707058 PMC3972767

[21] Sih A Bell A Johnson JC. Behavioral syndromes: an ecological and evolutionary overview. Trends Ecol Evol. (2004) 19:372–8. 10.1016/j.tree.2004.04.009 16701288

[22] Weary DM Huzzey JM von Keyserlingk MAG. Board-invited review: using behavior to predict and identify ill health in animals. J Anim Sci. (2009) 87:770–7. 10.2527/jas.2008-1297 18952731

[23] Rosenberger K Costa JHC Neave HW von Keyserlingk MAG Weary DM. The effect of milk allowance on behavior and weight gains in dairy calves. J Dairy Sci. (2017) 100:504–12. 10.3168/jds.2016-11195 27865513

[24] Svensson C Jensen MB. Short communication: identification of diseased calves by use of data from automatic milk feeders. J Dairy Sci. (2007) 90:994–7. 10.3168/jds.S0022-0302(07)71584-9 17235177

[25] Neave HW Costa JHC Weary DM von Keyserlingk MAG. Personality is associated with feeding behavior and performance in dairy calves. J Dairy Sci. (2018) 101:7437–49. 10.3168/jds.2017-14248 29729921

[26] Appleby MC Weary DM Chua B. Performance and feeding behaviour of calves on *ad libitum* milk from artificial teats. Appl Anim Behav Sci. (2001) 74:191–201. 10.1016/S0168-1591(01)00171-X

[27] Miller-Cushon EK Bergeron R Leslie KE DeVries TJ. Effect of milk feeding level on development of feeding behavior in dairy calves. J Dairy Sci. (2013) 96:551–64. 10.3168/jds.2012-5937 23164223

[28] McGuirk SM Peek SF. Timely diagnosis of dairy calf respiratory disease using a standardized scoring system. Anim Health Res Rev. (2014) 15:145–7. 10.1017/S1466252314000267 25410122

[29] R Core Team. R: A Language and Environment for Statistical Computing (2021).

[30] Tolkamp BJ Allcroft DJ Austin EJ Nielsen BL Kyriazakis I. Satiety splits feeding behaviour into bouts. J Theoret Biol. (1998) 194:235–50. 10.1006/jtbi.1998.0759 9778436

[31] Howie JA Tolkamp BJ Avendaño S Kyriazakis I. A novel flexible method to split feeding behaviour into bouts. Appl Anim Behav Sci. (2009) 116:101–9. 10.1016/j.applanim.2008.09.005

[32] Tolkamp BJ Kyriazakis I. To split behaviour into bouts, log-transform the intervals. Anim Behav. (1999) 57:807–17. 10.1006/anbe.1998.1022 10202089

[33] Bürkner P-C. Advanced Bayesian multilevel modeling with the R Package brms. R J. (2018) 10:395. 10.32614/RJ-2018-017

[34] Peterson RA Cavanaugh JE. Ordered quantile normalization: a semiparametric transformation built for the cross-validation era. J Appl Stat. (2020) 47:2312–27. 10.1080/02664763.2019.1630372 PMC9042069 35707424

[35] Mitchell DJ Beckmann C Biro PA. Understanding the unexplained: the magnitude and correlates of individual differences in residual variance. Ecol Evol. (2021) 11:7201–10. 10.1002/ece3.7603 34188806 PMC8216950

[36] Whishaw IQ Dringenberg HC Comery TA. Rats (Rattus norvegicus) modulate eating speed and vigilance to optimize food consumption: effects of cover, circadian rhythm, food deprivation, and individual differences. J Comp Psychol. (1992) 106:411–9. 10.1037/0735-7036.106.4.411 1451425

[37] Nielsen BL Lawrence AB Whittemore CT. Effect of group size on feeding behaviour, social behaviour, and performance of growing pigs using single-space feeders. Livestock Prod Sci. (1995) 44:73–85. 10.1016/0301-6226(95)00060-X

[38] Cellier M Duvaux-Ponter C Nielsen BL. Inter- and intra-individual variability of feeding behaviour in group housed dairy goats. Appl Anim Behav Sci. (2021) 234:105167. 10.1016/j.applanim.2020.105167

[39] Giger-Reverdin S Duvaux-Ponter C Sauvant D Friggens NC. Repeatability of traits for characterizing feed intake patterns in dairy goats: a basis for phenotyping in the precision farming context. Animal. (2020) 14:1083. 10.1017/S1751731119002817 31769385 PMC7163394

[40] Nielsen BL. On the interpretation of feeding behaviour measures and the use of feeding rate as an indicator of social constraint. Appl Anim Behav Sci. (1999) 63:79–91. 10.1016/S0168-1591(99)00003-9

[41] Quimby WF Sowell BF Bowman JGP Branine ME Hubbert ME Sherwood HW. Application of feeding behaviour to predict morbidity of newly received calves in a commercial feedlot. Can J Anim Sci. (2001) 81:315–20. 10.4141/A00-098

[42] Knauer W Godden S Dietrich A Hawkins D James R. Evaluation of applying statistical process control techniques to daily average feeding behaviors to detect disease in automatically fed group-housed preweaned dairy calves. J Dairy Sci. (2018) 101:8135–45. 10.3168/jds.2017-13947 30007809

[43] Dingemanse NJ Réale D. Natural selection and animal personality. Behaviour. (2005) 142:1159–84. 10.1163/156853905774539445

[44] Neave HW Costa JHC Benetton JB Weary DM von Keyserlingk MAG. Individual characteristics in early life relate to variability in weaning age, feeding behavior, and weight gain of dairy calves automatically weaned based on solid feed intake. J Dairy Sci. (2019) 102:10250–65. 10.3168/jds.2019-16438 31477284

[45] Harris SM Descamps S Sneddon LU Bertrand P Chastel O Patrick SC. Personality predicts foraging site fidelity and trip repeatability in a marine predator. J Anim Ecol. (2020) 89:68–79. 10.1111/1365-2656.13106 31541578 PMC7004082

[46] Coppens CM Boer SF de Koolhaas JM. Coping styles and behavioural flexibility: towards underlying mechanisms. Philos Transac R Soc B Biol Sci. (2010) 365:4021–8. 10.1098/rstb.2010.0217 21078654 PMC2992750

[47] Jolles JW Briggs HD Araya-Ajoy YG Boogert NJ. Personality, plasticity and predictability in sticklebacks: bold fish are less plastic and more predictable than shy fish. Anim Behav. (2019) 154:193–202. 10.1016/j.anbehav.2019.06.022

[48] van Dixhoorn IDE de Mol RM van der Werf JTN van Mourik S van Reenen CG. Indicators of resilience during the transition period in dairy cows: a case study. J Dairy Sci. (2018) 101:10271–82. 10.3168/jds.2018-14779 30243630

[49] Poppe M Veerkamp RF van Pelt ML Mulder HA. Exploration of variance, autocorrelation, and skewness of deviations from lactation curves as resilience indicators for breeding. J Dairy Sci. (2020) 103:1667–84. 10.3168/jds.2019-17290 31759590

[50] von Keyserlingk MAG Brusius L Weary DM. Competition for teats and feeding behavior by group-housed dairy calves. J Dairy Sci. (2004) 87:4190–4. 10.3168/jds.S0022-0302(04)73563-8 15545382

[51] Sih A Cote J Evans M Fogarty S Pruitt J. Ecological implications of behavioural syndromes. Ecol Lett. (2012) 15:278–89. 10.1111/j.1461-0248.2011.01731.x 22239107

[52] Dammhahn M Dingemanse NJ Niemelä PT Réale D. Pace-of-life syndromes: a framework for the adaptive integration of behaviour, physiology and life history. Behav Ecol Sociobiol. (2018) 72:1–8. 10.1007/s00265-018-2473-y

[53] Stamps JA. Growth-mortality tradeoffs and ‘personality traits’ in animals. Ecol Lett. (2007) 10:355–63. 10.1111/j.1461-0248.2007.01034.x 17498134

[54] David M Auclair Y Cézilly F. Assessing short- and long-term repeatability and stability of personality in captive zebra finches using longitudinal data. Ethology. (2012) 118:932–42. 10.1111/j.1439-0310.2012.02085.x

[55] Kluen E Brommer JE. Context-specific repeatability of personality traits in a wild bird: a reaction-norm perspective. Behav Ecol. (2013) 24:650–8. 10.1093/beheco/ars221

[56] Buczinski S L Ollivett T Dendukuri N. Bayesian estimation of the accuracy of the calf respiratory scoring chart and ultrasonography for the diagnosis of bovine respiratory disease in pre-weaned dairy calves. Prev Vet Med. (2015) 119:227–31. 10.1016/j.prevetmed.2015.02.018 25794838

[57] Timsit E Dendukuri N Schiller I Buczinski S. Diagnostic accuracy of clinical illness for bovine respiratory disease (BRD) diagnosis in beef cattle placed in feedlots: a systematic literature review and hierarchical Bayesian latent-class meta-analysis. Prev Vet Med. (2016) 135:67–73. 10.1016/j.prevetmed.2016.11.006 27931931

[58] Timsit E Assié S Quiniou R Seegers H Bareille N. Early detection of bovine respiratory disease in young bulls using reticulo-rumen temperature boluses. Vet J. (2011) 190:136–42. 10.1016/j.tvjl.2010.09.012 20947394

[59] Araya-Ajoy YG Dingemanse NJ. Characterizing behavioural ‘characters’: an evolutionary framework. Proc R Soc B Biol Sci. (2014) 281:20132645. 10.1098/rspb.2013.2645 24335984 PMC3871316

[60] Santostefano F Wilson AJ Niemelä PT Dingemanse NJ. Behavioural mediators of genetic life-history trade-offs: a test of the pace-of-life syndrome hypothesis in field crickets. Proc R Soc B Biol Sci. (2017) 284:20171567. 10.1098/rspb.2017.1567 28978731 PMC5647303

