# Supplementary material for: Repeatability and Predictability of Calf Feeding Behaviors—Quantifying Between- and Within-Individual Variation for Precision Livestock Farming
Source: Front Vet Sci. 2022 Mar 31;9:827124. doi: 10.3389/fvets.2022.827124 (PMC9009244; doi:10.3389/fvets.2022.827124)
Supplement: Supplementary file 1 [file Table_1.DOCX]

**
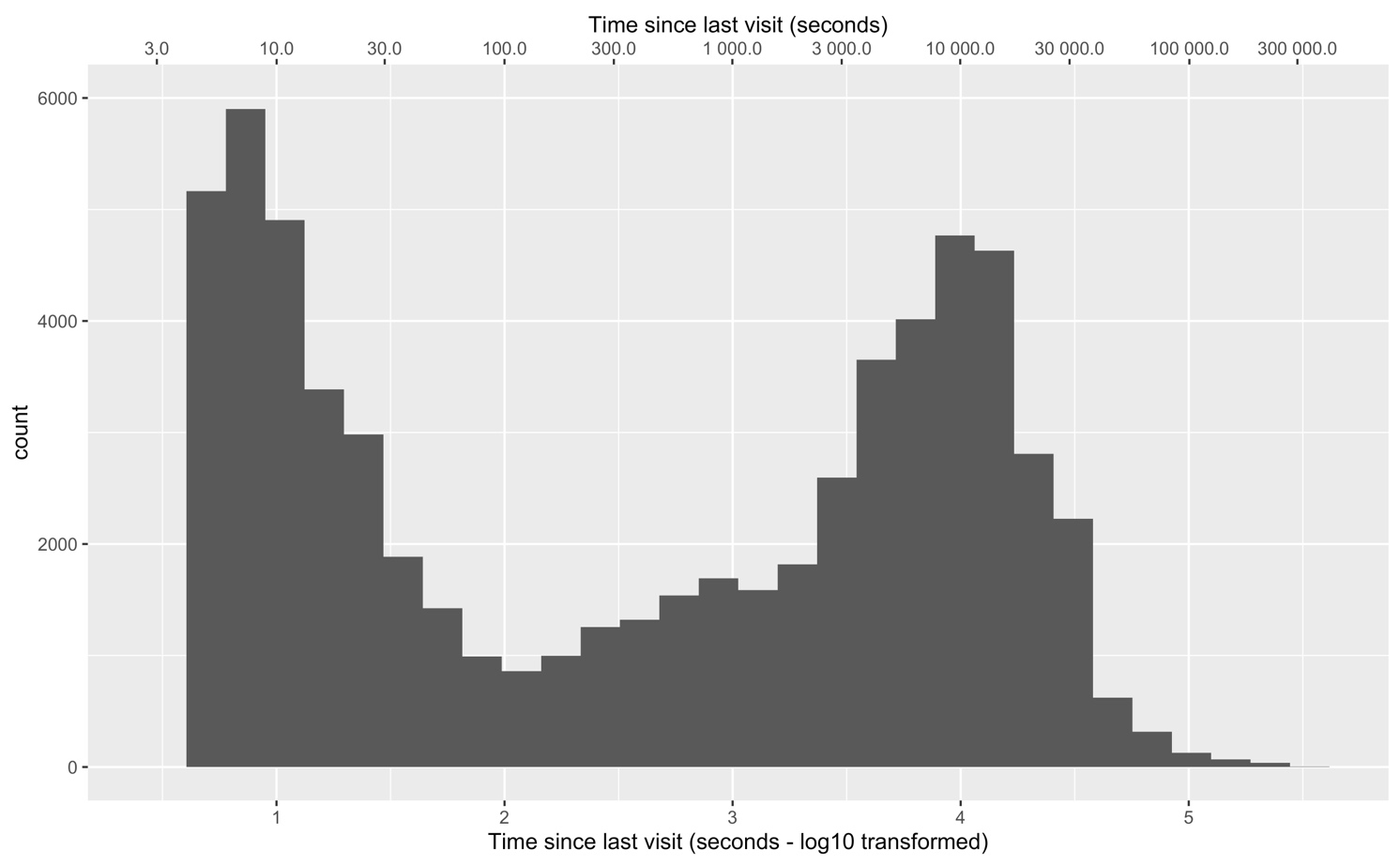
**

**Figure 1. Log 10 transformed time interval between consecutive visits**

Three log10 distributions with intersections of 10^2 (approximately 100 seconds) and 10^3.2 (approximately 1600 seconds) can be seen in the figure above. Where the same calf had visits to the feeder that were separated by a duration equal to or less than 100 seconds these visits were considered as the same meal. Visits that were separated by more than 100 seconds were considered as separate meals.

| **Correlations between behavioural type and residual intra-individual variation (rIIV) estimates** | | **Feeding Rate** | | **Total Meals** | | **Meal Size** | |
| --- | --- | --- | --- | --- | --- | --- | --- |
|  |  | **Behavioural Type** | **rIIV** | **Behavioral Type** | **rIIV** | **Behavioral Type** | **rIIV** |
| **Feeding Rate** | **Behavioral Type** |  | 0.09  [- 0.25 – 0.39] | **0.29**  **[0.00 – 0.54]** | - 0.11  [- 0.51 – 0.30] | -0.06  [- 0.50 - 0.39] | -0.14  [- 0.64 – 0.44] |
|  | **rIIV** |  |  | 0.07  [-0.26 – 0.39] | 0.24  [- 0.22 – 0.65] | -0.39  [- 0.78 – 0.08] | 0.25  [- 0.39 – 0.75] |
| **Total Meals** | **Behavioral Type** |  |  |  | 0.17  [- 0.23 – 0.55] | 0.07  [- 0.38 – 0.51] | - 0.11  [- 0.63 – 0.48] |
|  | **rIIV** |  |  |  |  | 0.04  [- 0.52 – 0.57] | 0.15  [- 0.51 – 0.72] |
| **Meal Size** | **Behavioral Type** |  |  |  |  |  | -0.40  [- 0.87 – 0.39] |
|  | **rIIV** |  |  |  |  |  |  |

**Table 1. Results of between variable correlations for behavioral types and residual intra-individual variation - estimates from multi-variate model.**
